# A Review on AA 6061 Metal Matrix Composites Produced by Stir Casting

**DOI:** 10.3390/ma14010175

**Published:** 2021-01-01

**Authors:** Ansar Kareem, Jaber Abu Qudeiri, Asarudheen Abdudeen, Thanveer Ahammed, Aiman Ziout

**Affiliations:** Mechanical Engineering Department, College of Engineering, United Arab Emirates University, Al Ain 15551, UAE; 201990209@uaeu.ac.ae (A.K.); 201990133@uaeu.ac.ae (A.A.); 201870099@uaeu.ac.ae (T.A.); ziout@uaeu.ac.ae (A.Z.)

**Keywords:** AA6061 alloy, fabrication methods, stir casing, metal matrix composites, reinforcements, microstructure, properties

## Abstract

In recent years, many alloys as well as composites of aluminium were developed for enhanced material performance. AA 6061 is an aluminium alloy that has extensive applications due to its superior material characteristics. It is a popular choice of matrix for aluminium matrix composite (AMC) fabrication. This study provides a review on AA 6061 aluminium alloy matrix composites produced through the stir-casting process. It focusses on conventional stir-casting fabrication, process parameters, various reinforcements used, and the mechanical properties of the AA 6061 composites. Several research studies indicated that the stir-casting method is widely used and suitable for fabricating AA 6061 composites with reinforcements such as SiC, B_4_C, Al_2_O_3_, TiC, as well as other inorganic, organic, hybrid, and nanomaterials. The majority of the studies showed that an increase in the reinforcement content enhanced the mechanical and tribological properties of the composites. Furthermore, hybrid composites showed better material properties than single reinforcement composites. The usage of industrial and agricultural residues in hybrid composites is also reported. Future studies could focus on the fabrication of AA 6061 nanocomposites through stir casting and their material characterisation, since they have great potential as advanced materials.

## 1. Introduction

Aluminium alloys are the predominant nonferrous metal used in various applications due to their plentiful desirable material properties. As a result of the extensive studies conducted, numerous alloys of aluminium have been created with an objective to improve the specific required material properties. Composites are the multiphase materials that consist of matrix and reinforcement, which were developed to fulfil the ever-increasing demand of attractive engineering materials. Generally, composites exhibit excellent thermal properties and outstanding mechanical characteristics including higher strength, hardness, fracture toughness, and better resistance to wear and corrosion. These advantageous characteristics led to the increased use of composite materials in industrial applications [1]. Composites are classified according to the type of matrix material present in it. They are categorised into three major types as polymer matrix composites (PMCs), ceramic matrix composites (CMCs), and metal matrix composites (MMCs). MMCs are the most widely used type of composites in industrial applications, because of their various advantages relative to PMCs and CMCs [2].

In MMCs, matrix material will be a metal. It is the continuous phase of composite and functions as a binder that surrounds the reinforcement. The metal matrix transmits and distributes the load to the reinforcement, which is the dispersed phase [3]. Aluminium matrix composites (AMCs) are having pure aluminium or its alloy as the matrix, and they are being increasingly utilised in industrial applications owing to their remarkable mechanical, material, and tribological characteristics. This led to the development of AMCs with every possible aluminium alloy as matrix, incorporated with various reinforcement materials to achieve the specific desired properties. AMCs can be manufactured through numerous methods according to their end use. Since the 1930s, aluminium alloys have been the preferred material for the production of airplane parts [4]. The AA 6XXX aluminium alloy series in which silicon and magnesium are the principal alloying elements are gaining particular interest in the aviation and automotive industries. The remarkable strength to weight ratio offered by this series of alloy along with their better formability, weldability, resistance to corrosion and wear, and low cost made it a potential material for the manufacturing of light weight vehicles [5]. AA 6061 is one among the most popular alloy in 6XXX series, being used as matrix material in numerous AMCs because of the possibility to alter the composite strength through suitable heat treatment.

Composite materials developed with enhanced characteristics acknowledged a great deal of attention in multiple areas such as aviation, automotive, military, and other manufacturing industries because of their distinct features and superior quality compared to their base materials [6]. A combination of the most important properties of matrix and reinforcements is the significant benefit achieved through composite production. High strength materials are fabricated by the combination without compromising the excellent ductility and density of alloys. The possibility of adding high strength particles as reinforcements also helps to overcome any disadvantages of matrix materials [7]. Reinforcement addition in an aluminium matrix is reported to cause improvement in the tensile strength, compressive strength, impact strength, and hardness of the composite. Usually, the wear resistance of AMCs is also higher than that of unreinforced aluminium or aluminium alloys [8,9]. Materials of various kinds are utilised as reinforcements in the manufacturing of AMCs. They can be used in the form of particles, whiskers, short fibers, and continuous fibers. Compared to the other types, particle reinforcements have better isentropic properties that makes them capable of distributing uniformly in the matrix phase [10]. Hence, they are preferred in the AMC fabrication and are used mostly in the automobile components manufacturing due to its superior tribological properties. Ceramic, synthetic, industrial waste, and agro waste reinforcement particles having a micro or nano size can be effectively blended with matrix material to produce AMCs [11].

Composites have been ascertained as a potential alternative to conventional materials, but there are still some hurdles in the research and development of composites. The key objective of composite production is to achieve improved material characteristics, which depends upon many factors such as fabrication route, process parameters, constituent materials, and composition. Suitable materials as well as fabrication methods must be selected along with optimum process parameters for achieving the desired properties. A broad range of manufacturing techniques has been explored for MMCs, which include liquid-state methods and solid-state methods [12,13,14]. Due to the salient features of the stir-casting method, it is the most popular fabrication route employed commercially [15,16]. The simplicity and flexibility of the process made it an economical method suitable for large-scale fabrication. Complex profiled MMCs can be produced using stir casting without damaging the reinforcement particles [17]. Attention should be given to achieve a uniform distribution of reinforcement materials while fabricating composites through the stir-casting method [18].

AA 6061 is the most versatile alloy among the aluminium 6XXX alloy series, and the AA 6061 composites are mostly produced by the stir-casting technique. Although many researchers have studied numerous AA 6061 composites and their fabrication, there is no comprehensive review on AA 6061 stir-cast composites to the best of the authors’ knowledge. The main objective of the paper is to review the various possible MMCs fabricated through stir casting with AA 6061 as the matrix material. The authors aim to discuss the effect of various reinforcements in the AA 6061 matrix and the feasibility of the stir-casting process for these materials.

## 2. AA 6061 Aluminium Alloy

Currently, different kinds of aluminium alloys are commercially available, and each one has its own unique advantage and applications. This paper focusses on AA 6061 aluminium alloys that are heat treatable, can be appreciably strengthened, and are used for various applications in which strength, weldability, and corrosion resistance are inevitable [19,20]. The AA 6061 alloy composition is shown in Table 1 below [21].

The base alloy has a tensile strength of 115 MPa, rockwell hardness of 30 HRB, and elastic modulus of 70–80 MPa. AA 6061 alloys are primarily used in automobile and aviation sectors for manufacturing light weight parts. A wide variety of reinforcements have been used to fabricate the metal matrix composite (MMC) using the AA 6061 matrix, which include the compounds such as SiC, B_4_C, Al_2_O_3_, TiC, Si_3_N_4_, BN, ZrO_2_, and so on. Several nanocomposites are also produced in larger scale recently, with the AA 6061 alloy matrix [22].

## 3. Stir Casting and AMC

In the past decade, an extensive range of methods has been developed for producing MMCs. The mechanical properties and production cost of the composite significantly depend upon the type of fabrication method involved. These fabrication methods can be classified into solid- and liquid-state processing based on the state of the metal matrix in primary process treatment. Moreover, there are also other techniques such as compocasting, rheocasting, in situ fabrication, and spray deposition, which involve a semi-solid condition of the matrix, but these are not as popular as the solid- or liquid-state techniques [23]. In solid-state fabrication, the bonding of a matrix with reinforcements occurs as a result of the mutual diffusion arising between them in solid state at higher levels of temperature and pressure. Liquid state fabrication involves the dispersion of reinforcements in the molten matrix, followed by its solidification, either through infiltration or casting methods [24]. These methods are cost effective compared to solid-state methods. Stir casting is the most popular and commercially used technique in liquid-state processing, since it is economical compared to other manufacturing techniques. It also provides fairly homogenous dispersion of reinforcements in matrix, better wettability, and reduced porosity [25].

### 3.1. Stir-Casting Process Outline

**Figure 1:**
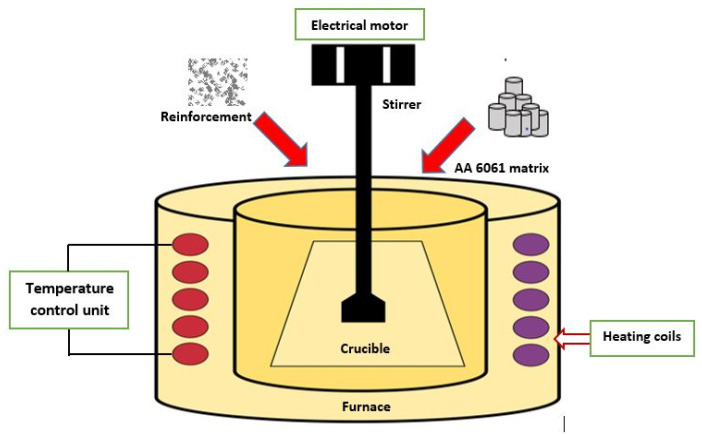
Schematic diagram of stir-casting method of AA 6061 composites.

Stir-casting primarily involves the mixing of a dispersed phase in matrix phase, which is facilitated using a stirring mechanism. Electrical energy is often used to energise the stir-casting furnace, and electrical resistance heating is the commonly used method of heat generation. The process comprises of heating the matrix placed in the crucible, up to its melting point. Crucible is made as chemically inert to the matrix and reinforcements. Preheating of the reinforcements is often carried out to improve the mixing between the materials. Mixing takes place in molten condition, and an inert condition may be kept during the stirring and pouring of charge so as to reduce the chances of casting defects. Particulate reinforcements are usually fed through an injection gun in order to reduce the possibility of gas entrapment. Propeller blades of the stirrer are attached to a shaft connected with the output of the electrical motor, which imparts rotational motion. Vertical motion of the stirrer can be effectively controlled through a lead screw arrangement powered by another electrical motor. Stepper motors are commonly used for varying the rotational speed of the stirrer [26]. For achieving homogenous mixture through this process, wettability between the matrix and reinforcement should be proper [27]. Schematic diagram of the stir-casting process of AA 6061 composites is shown in Figure 1.

### 3.2. Stir-Casting Process Parameters

Several stir-casting process parameters have a substantial impact on determining the characteristics of AMCs. Nevertheless, process parameters such as the reinforcement size, speed of stirrer, stirring time, stirrer blade design, and melt temperature are found to have a maximum impact. Conveniently, those parameters can be easily altered without any additional effort and expense throughout the process. Hence, the selection of process variables is of considerable importance [28]. The significant process parameters in stir casting are given below:

Reinforcement size: The strength of the material fabricated through stir casting is prominently influenced by the size of the reinforcement. Often, composites with smaller reinforcement size exhibit superior mechanical properties. Yehia M. Youssef et al. [29] fabricated Al-10Sb cast aluminium alloy–SiC composites. Particle sizes of SiC used in the process were 115, 225, and 350 μm; 3%, 5%, and 9% weight fractions of SiC were selected for reinforcing the matrix. On material characterization, the composite with the finest reinforcement particles (115 μm size) with 9 wt % SiC exhibited maximum improvement of mechanical properties. It points toward the fact that the strengthening effect is maximum when finer particles of reinforcements are added. Palash Poddar et al. [30] fabricated AZ91D alloy-SiC AMC through the stir-casting method. The volume fraction of SiC was 15% and reinforcements with average particle sizes of 15 and 150 µm were added. It was reported that a reduction in average grain size occurred when reinforcements were added. Composite reinforced with 15 µm particles possessed much smaller grain size than composites with 150 µm particles. Grain refinement was accelerated with finer particulates. AZ91D alloy-15 µm SiC composites also had superior mechanical properties compared to composites with 150 µm SiC.

Stirring speed and stirring time: The viscous nature of the molten matrix in AMC plays an essential part in controlling the quality of reinforcement distribution. Higher viscosity restricts the smooth movement of reinforcement particles during stirring, which is not preferred. On the other hand, lower viscosity also should be avoided, since the suspension and holding of particles is not effective in this condition. Increasing the speed of stirring can increase the inter-particle distance. The stirrer speed is dependent on the stirrer blade profile. Thus, an exact prediction of optimum speed is not always possible. Properties of the composites will be maximum when a uniform dispersion of reinforcements occurs. Higher levels of inter-particle distance and homogeneity in distribution can be achieved at higher stirring time. However, stirring time varies according to the shape of the blade. So, specifying the optimum value accurately is not practical [23]. J. Jebeen Moses et al. [31] reported that different stir-casting parameters such as stirring speed, stirring time, blade angle, and casting temperature considerably influenced the mechanical properties of AMC. A combination of larger or smaller values of these process variables led to poor ultimate tensile strength (UTS). This was due to the porosity formation, grouping, and segregation of reinforcements at the grain boundaries. The least porous and efficient casting products with uniformly dispersed reinforcements were obtained when an intermediate range of process variables was selected. Thus, it is preferred over extreme levels of parameters.

Melt temperature: Wettability of the melt can be enhanced by providing high melt temperature. However, high temperature is not desirable always, since it lowers the viscosity of melt. A low temperature of melt favors particle agglomeration in it. Hence, an optimum intermediate value of temperature is desirable for better properties.

Stirrer blade design: Zirconia is often used as a coating in stainless steel stirrer blades, since it can prevent reactions between stainless steel and aluminium alloys at higher temperatures. Therefore, a zirconia coating is highly recommended in the stir casting of AA 6061 MMCs. An impeller design should aid in the creation of a vortex so that perfect mixing of a melt can be accomplished [23].

### 3.3. AA 6061 AMC Fabrication

H. Karakoç et al. [32] fabricated AA 6061-B4C composites using powder metallurgy, which is the predominant solid-state processing technique. The MMC was reinforced with weight fractions of 5%, 10%, 15%, and 20% of B_4_C. The matrix structure had uniform reinforcement particle distribution and the resulted composite had good relative density, hardness, and tensile strength. M. Dhanashekar et al. [33] successfully made an AA 6061/SiC composite through powder metallurgy, and mechanical properties were investigated. Microstructural evaluation revealed that the particles were uniformly distributed and a strong bond existed between the matrix and reinforcements. As the weight percentage of the SiC reinforcement increases, the hardness, density, and compression strength of the composites were also increased. Researchers have produced AA 6061 composites with different types of reinforcements, but the majority of the composites were fabricated through stir-casting route, which is the most popular liquid-state processing method. The process involves a conventional metal processing route, and production cost is reduced. The feasibility of fabricating large-sized composites also makes it an attractive method of production. For achieving the desired characteristics of MMC, the reinforcement distribution in the matrix material should be homogenous, the wettability between them must be optimised, chemical inertness should prevail between them, and porosity needs to be minimum [18,34]. Hence, by properly controlling the processing conditions and weight fraction of reinforcement, AA 6061 composites with enhanced characteristics can be satisfactorily produced.

## 4. AA 6061 Composites Developed through the Stir-Casting Route

Researchers have developed an extensive set of AA 6061 composites using organic as well as inorganic reinforcements. The primary purpose of the dispersed phase is to bind the matrix in proper manner so as to enhance the properties of base materials. Generally, reinforcement weight composition in the composite ranges from 5 wt % to 30 wt % as that of AA 6061 alloy. Several types of reinforcements are made to mix together and used in composite production as hybrid reinforcement, which further improves the properties. Nano AA 6061 composites are also gaining popularity among researchers, in which nano-sized particles are used as reinforcements. This section discusses the research studies based on the different kinds of reinforcements used in the AA 6061 composites.

In this section, studies related to the AA 6061 composites fabricated through the stir-casting method are classified according to the reinforcements used and are as follows:

1. AA 6061-SiC composites
2. AA 6061-B_4_C composites
3. AA 6061-Al_2_O_3_ composites
4. AA 6061-TiC composites
5. AA 6061 composites with other reinforcements (other than SiC, B_4_C, Al_2_O_3_, TiC)
6. AA 6061-hybrid composites
7. AA 6061-nanocomposites

Using this classification scheme, Table 2 lists the studies of AA 6061 composites with different reinforcement types.

### 4.1. AA 6061-SiC Composites

**Figure 2:**
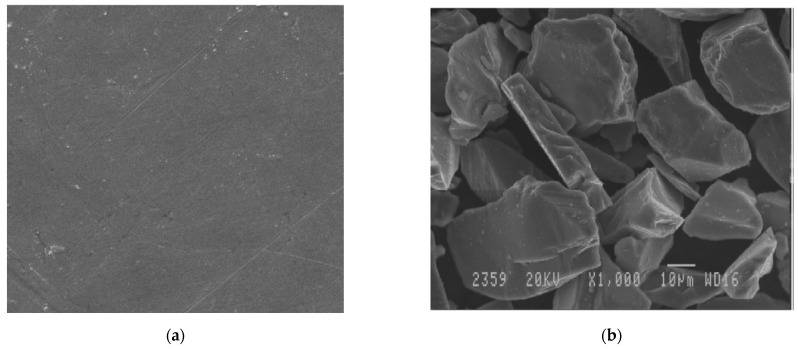
(**a**) Scanning electron micrograph of AA 6061 alloy; (**b**) Scanning electron micrograph of silicon carbide powder [35].

Silicon carbide (SiC), which is also known as carborundum, has good mechanical and thermal characteristics. The density of SiC is comparable to that of aluminium. SiC-reinforced aluminium alloy composites are the typical choice of material for various functionalities in aviation, defense, structural, and manufacturing industries, owing to their exceptional mechanical and tribological properties [77]. G.B. Veeresh Kumar et al. [35] successfully adopted the stir-casting route for the fabrication of AA 6061-SiC composites, which constitutes the reinforcement contents from 2 to 6 wt %. Figure 2a shows the microstructure of AA 6061 alloy when observed under scanning electron microscope. The scanning electron micrograph of silicon carbide powder is also shown in Figure 2b, which indicates the morphology of the reinforcement material. It shows that the typical SiC particulates consists of round and angular grains with sharp cornered morphology.

**Figure 3:**
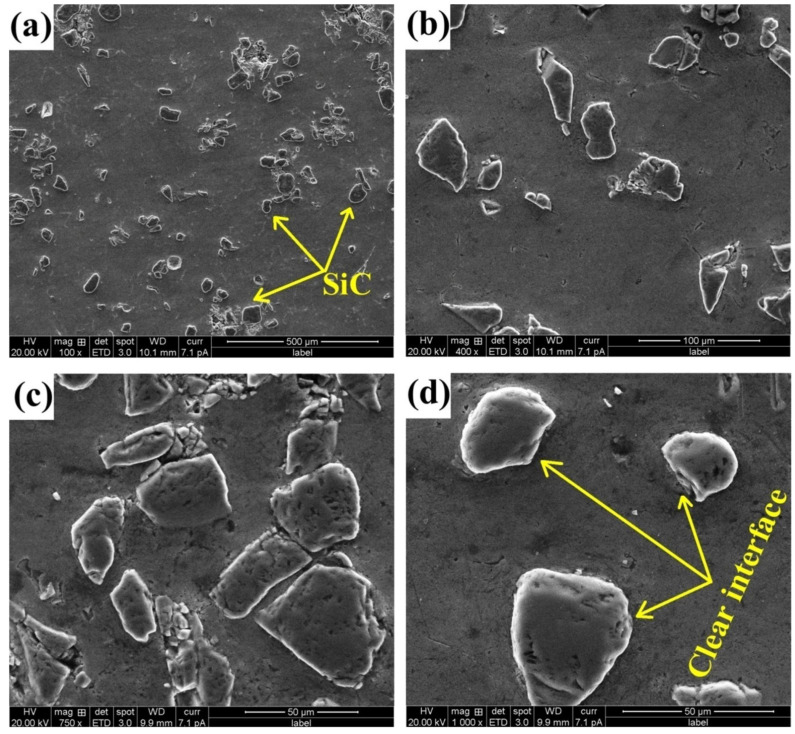
SEM images of AA 6061-15 vol.% SiC at magnification (**a**) 100×; (**b**) 400×; (**c**) 750×; (**d**) 1000× [36].

A uniform distribution of particulates in the matrix was revealed by microstructural examination, which indicated that the process was flawless. As the concentration of SiC particles increased in the matrix, composite properties such as hardness, UTS, and resistance to wear also showed improvement. Density was also superior to that of the virgin alloy. J. Jebeen Moses et al. [36] attempted to fabricate AA 6061 MMC with silicon carbide particulates of 5, 10, and 15 percentages of weight fraction. Optical microscopic and scanning electron microscopic analyses revealed that the SiC distribution was homogenous and led to grain refinement. Figure 3 shows the SEM images of AA 6061-SiC composite with 15% volume fraction.

From Figure 3, it is evident that SiC particulates were homogenously distributed in the aluminium alloy matrix. No segregation of SiC particles were found along the grain boundaries. Distribution of particles was observed to be intra-granular, in which the majority of the particles locate inside the grains. This distribution is preferred in AMCs to have better mechanical and tribological properties. Adequate mechanical stirring action caused the reinforcements to be effectively dispersed in the molten matrix. SiC particles were thermodynamically stable, and there were no pores or voids around them. SiC particles resisted aluminium grain growth and resulted in nucleation sites growth, which led to the formation of finer grains. Figure 3d represents the higher magnification (1000×) of the AA 6061-15% SiC composite. It indicates that the interface between the matrix and dispersed phase is clear, since any reaction products were not presented. The AA 6061/15 wt % composite showed 133.33% higher microhardness and 65.2% higher ultimate shear strength compared to unreinforced AA 6061 alloy.

AA 6061 alloy was reinforced with 0–4 wt % of SiC particulates through stir casting by S. Sivananthan et al. [37], and the impact of reinforcement on mechanical characteristics of the composite was studied. For every weight fraction of SiC, the resulting composites exhibited improved properties than base alloy. In the case of 4 wt % SiC composites, the hardness, tensile strength, and compression strength increased by 25%, 25.6%, and 12%, respectively, when compared to that of AA 6061 alloy. Thus, hard ceramic particles of reinforcements led to this improvement. Due to its outstanding material properties compared with aluminium alloys, these composites have vast applications in aviation and automobile industries.

The electromagnetic stir-casting method is utilised to fabricate AA 6061/SiC composite with varied weight fractions (0%, 1%, 2%, 3%, and 5%) [38]. The uniform distribution of SiC particles without any agglomeration was observed using scanning electron microscopy. The density, hardness, and tensile strength of composites showed improvement as compared to the base alloy. In electromagnetic stir casting, a three-phase induction motor is used to generate an electromagnetic field that is strong enough to stir the molten matrix after the addition of reinforcement particulates. The molten material rotates continuously by the electromagnetic field until solidification. This stirring action would lead to the uniform distribution of reinforcement particles and subsequent improvement in mechanical properties [78,79].

### 4.2. AA 6061-B_4_C Composites

Boron carbide (B_4_C) is a black solid having metallic shining and is known as one of the hardest ceramic materials found in earth [80]. It is an attractive reinforcement material due to its exceptional thermal and chemical stability. Moreover, it has a lower density of 2.52 g/cm^3^ and higher hardness (HV) of 30 GPa, compared to Al_2_O_3_ and SiC. It is used to manufacture military tanks and bullet proof jackets. Thus, B_4_C-reinforced AMCs fabricated through the low-cost stir casting method have attained greater attractiveness [81,82]. K. Kalaiselvan et al. [39] produced AA 6061-B_4_C composites successfully by stir casting with various weight percentage (4, 6, 8, 10, and 12 wt %) of reinforcement. They reported that B_4_C particles in the composite were homogeneously dispersed. For enhancing the wettability of B_4_C particles with aluminium melt, K_2_TiF_6_ flux was added. Reaction of the flux on the molten surface generated heat in the area close to the interface and due to the local elevation of temperature, particle incorporation into the melt improved, and enhanced bonding was achieved. Efficient stirring with suitable process parameters led to the homogenous spreading of reinforcements. The hardness and tensile strength of the composites were increased along with the weight percentage of B_4_C particles.

Composites with 5 wt % and 10 wt % of B_4_C particles were fabricated by B. Ravi et al. [40] and revealed that the dispersion of particles took place homogenously in the AA 6061 matrix due to effective stirring and appropriate process variables. B_4_C particles initiated the increment in nucleation sites during solidification, and as a result, grain size was decreased. Hardness was increased due to the existence of hard reinforcement particles on the surface, which resisted plastic deformation. Since improved interfacial bonding occurred between the matrix and reinforcement, load was effectively transferred and distributed from the matrix to the reinforcement, and it increased the UTS of the composite. Bhujanga D. P. et al. [41] examined the wear behavior of AA 6061-B_4_C composites and found that capability of wear resistance improved with the increase in weight fraction of B_4_C. Substantial improvement was observed due to the incorporation of hard ceramic particulates on ductile AA 6061 matrix. B. Manjunatha et al. [42] illustrated that the extrusion process can be carried out after the fabrication of AA 6061-B_4_C composite through stir casting. Extrusion process further improved the particle distribution, helped reduce the particle size, and eliminated the casting defects. Heat treatment can be also carried out to improve the mechanical properties. The stir-casting technique is even capable of fabricating AA 6061−B_4_C composites with very high B_4_C content up to 31% of weight fraction [43]. Vacuum stirring and the progressive addition of reinforcement were needed to incorporate a high content of B_4_C into matrix. SEM analysis revealed the uniform distribution of the reinforcement. The AA 6061−31% B_4_C composite had a UTS of 340 MPa. Thus, stir casting can be regarded as the most promising technique in the fabrication of AA 6061−B_4_C composites.

### 4.3. AA 6061-Al_2_O_3_ Composites

**Figure 4:**
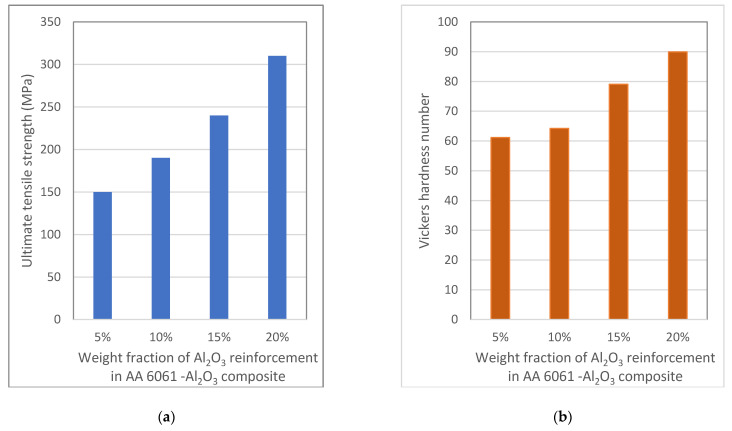
(**a**) Variation of ultimate tensile strength (UTS) of AA 6061-Al_2_O_3_ composite with increased content of Al_2_O_3_; (**b**) Variation of Vickers hardness value of AA 6061-Al_2_O_3_ composite with increased content of Al_2_O_3_ (Data from [45]).

Among the numerous reinforcement materials of AMCs, aluminium oxide (Al_2_O_3_) is the most often used reinforcement next only to silicon carbide, as it possesses excellent interfacial compatibility [83]. Al_2_O_3_ is a hard ceramic material with moderate density (3.97 g/cm^3^) and high thermal expansion coefficient [44]. The resulting set of composites also show similar results as discussed in previous sections. AA 6061/Al_2_O_3_ composites were successfully developed using the stir-casting technique by Bhaskar Chandra Kandpal and co-authors [45]. AA 6061 alloy was reinforced with 5%, 10%, 15%, and 20% of Al_2_O_3_. Microstructures obtained using SEM revealed a fairly homogenous distribution of reinforcement particles. The mechanical characterisation revealed that the strength and hardness were improved as the weight fraction of reinforcement increased from 5% to 20%. The variations of ultimate tensile strength and Vickers hardness value obtained for the different weight fractions of Al_2_O_3_ are shown in Figure 4a,b respectively. The tensile test and hardness test revealed that as the weight fraction increases, the UTS and hardness value also increases. The presence of hard ceramic reinforcement particulate improved the properties of the material. It was inferred that the mechanical properties of the AMCs were strongly related to the amount of reinforcement content.

### 4.4. AA 6061-TiC composites

Titanium carbide (TiC)-reinforced composites exhibit excellent bonding features especially with aluminium and were ascertained as an effective and promising reinforcement for enhanced material characteristics such as micro hardness, wear resistance, and compressive strength. TiC particulates are also accepted as a credible reinforcement for achieving greater corrosion resistance in AMCs [46]. S. Gopalakrishnan et al. [47] fabricated AA 6061-TiC particulate-reinforced composite through an enhanced stir casting method. In this method, magnesium was supplemented during stirring in order to improve the wettability. Argon gas was also used to prevent the reaction of molten matrix material with atmosphere. Defect-free composites with varied reinforcement content were fabricated. A substantial increase in the specific strength of the material was observed as the concentration of TiC was increased, which is attributed to the resistance offered by TiC during plastic deformation. Wear analysis using pin on disc machine revealed that the wear resistance was also improved in the AMC. Stir casting was also employed successfully in the production of an AA 6063 alloy-TiC composite, with a fair dispersion of TiC. It also showed a similar trend, having improvement in the density, hardness, and tensile strength of the composite [48]. M.S. Raviraj et al. [49] fabricated AA 6061-TiC MMC with 3 wt %, 5 wt %, and 7 wt % of TiC by the stir-casting method, without any flaws. TiC addition considerably refined the grain structure and increased the strength of the composites.

### 4.5. AA 6061 Composites with Other Reinforcements

Researchers have successfully fabricated AA 6061 composites with several other reinforcements than SiC, B_4_C, Al_2_O_3_, and TiC, using stir casting. AA 6061 matrix was reinforced with iron to form AMC through the stir-casting route [50]. The composite was reinforced with iron ore of 2%, 4%, and 6% weight fraction. Cast samples were heat treated by three stages, namely, solution treatment, quenching, and ageing. AA 6061 alloy was measured with an ultimate tensile strength of 173.3 N/mm^2^, while an AA 6061/6 wt % iron ore composite had 240.5 N/mm^2^ strength with a significant increase of 38%. Hardness was increased from 72 to 103 BHN when reinforcement changed from 0 to 6 wt % (45% increase). Scanning electron microscopy (SEM) photographs demonstrated that iron ore particles were uniformly distributed in the AA 6061 matrix, which revealed an excellent bond between the matrix and reinforcement.

Hematite is an oxide of iron (Fe_2_O_3_) found abundantly in nature. It is harder than pure iron but more brittle in nature. AA 6061-Fe_2_O_3_ composites were fabricated using the stir-casting method by adding the reinforcement in particulate form from 0 to 8 wt %, with an increment of 2 wt % [51]. Microstructural study revealed that a uniform distribution of reinforcement particles existed in all the composites developed. The grain boundary lines in the microstructure appeared as denser as the weight fraction of Fe_2_O_3_ increased, which indicated a significant improvement in hardness. The maximum increase of hardness and UTS was observed at 8 wt % of reinforcement. A 30% increment in hardness and 25% in UTS were obtained compared to the corresponding values of virgin alloy. A dry sliding wear test indicated that the wear resistance of the composite increased with an increase in the reinforcement weight fraction. When compared to the unreinforced alloy, the wear factor of the composite with 8% of hematite was lowered by 30–40%. The presence of harder hematite reinforcement imparted a hardness property to the composite and led to this effect.

AA6061-glass particulate AMC was developed using the stir-casting method [52]. Composites with different volume fractions of glass such as 3%, 6%, 9%, and 12% were fabricated. It was found that the hardness and tensile strength increased with a rise in glass content up to 9% volume fraction, but it decreased for the 12% glass composite due to the presence of agglomeration and pores. The tensile strength improved from 119 to 192 MPa. The addition of a reinforcement led to the increased number of dislocations, which restricted the movement of dislocations and thus increased the tensile strength of the composite.

Chethan K. N. et al. [53] studied the feasibility of using bamboo charcoal particulate as reinforcement in the AA 6061 matrix and its effect on mechanical properties. First, 2%, 4% and 6% weight fractions of bamboo charcoal were reinforced into matrix. Due to the presence of carbon in reinforcement particulates, the hardness value of the composites increased. The furnace cooled composite with 6 wt % bamboo charcoal showed a maximum hardness of 112 VHN, which is significantly higher than the hardness of AA 6061 alloy (105 VHN). However, the tensile strength decreased with the increase in bamboo charcoal content for all samples of composites.

A limited number of studies have been carried out regarding AMCs reinforced with molybdenum disulfide (MoS_2_) because of the high material cost and poor wetting of reinforcement. Being a robust material, MoS_2_ exhibits outstanding thermal as well as chemical stability, good strength, and low density of 2.52 g/cm^3^. Stir casting was performed for an AA 6061 alloy with different weight fractions (1%, 2%, 3%, 4%, and 5%) of MoS_2_. It was noted that the hardness was measured as 65 HV for 1 wt % MoS_2_. As the reinforcement content was gradually increased to 4 wt %, the hardness value also had a significant increment to 109 HV. Composites were heat treated, and those composites showed better hardness values than the as cast ones. The 4% of MoS_2_ addition resulted in the maximum hardness of the composite. The exact same pattern of results were obtained during yield strength and UTS measurements. Reinforcement addition beyond 4 wt % decreased the mechanical properties, which might be due to porosity or defects in stir casting [54].

An AA 6061 red-mud composite was fabricated using stir casting [55]. Heat treatment of the fabricated composites was performed to obtain better and uniform properties. Improved surface properties and reduced cracks were observed in heat-treated composites. SEM images demonstrated that red-mud particles had a fairly homogenous distribution in the matrix.

Rutile, being a natural form of TiO_2_, has better mechanical, tribological, and electrical properties as well as a lower thermal expansion coefficient. It was successfully reinforced with AA 6061 alloy matrix through the stir-casting method [56]. SEM analysis demonstrated the uniform distribution of rutile particulates in the matrix. The density and hardness of the composites were higher than those of AA 6061 alloy, owing to the inclusion of high density and harder rutile particles. For 1%, 2%, 3%, and 4% weight fractions of reinforcement addition, the hardness values of the composites increased by 15%, 24%, 36%, and 44%, respectively, and tensile strength was enhanced by 5%, 10%, 14%, and 7%, respectively, in comparison with the corresponding values of unreinforced alloy. It was reported that reinforcement addition in excess of a 3% weight fraction did not had an advantageous effect on the tensile strength of the composite due to the agglomeration and non-homogenous distribution of rutile particles.

Steel machining chips in powdered form, having a particle size of 40 to 60 microns, were used as reinforcement in AA 6061 matrix by Md Sumair et al. [57]. First, 5% and 10% weight fractions of chips were reinforced through the stir-casting method. The tensile strength and hardness of composites were reported as improved when compared to the properties of unreinforced matrix material. The wear index decreased as the weight fraction of steel chips was increased because steel chips are harder than aluminium, and they impart higher resistance when subjected to wear. Researchers were able to develop a better material with reliable and economical reinforcement by effectively utilising the industrial waste and hence enhancing sustainability.

It can be inferred that the AA 6061 alloy can be reinforced with various organic and inorganic materials through the stir-casting process to enhance the properties.

### 4.6. AA 6061 Matrix Hybrid Composites

Hybrid composites are manmade composite materials in which more than one type of reinforcement exists, which can be organic or inorganic materials. These composites are tailored to achieve superior characteristics, which are attributed to the existence of secondary reinforcements in the matrix. Custom-made hybrid materials of aluminium displayed excellent mechanical properties and coefficients of thermal expansion. For developing aluminium-based hybrid materials, the stir-casting method is extensively adopted. Material characteristics can be altered through the scientific optimisation of the quantity and type of constituent materials of hybrid composites [58]. The use of different reinforcements in hybrid MMC resulted in the increase of mechanical characteristics, but after a particular reinforcement level, it starts to decline. Hence, reinforcements should be added only up to a specific weight fraction; otherwise, there is no point in its addition. It can be inferred that this is due to the increased porosity and agglomeration at a higher reinforcement content and the optimum weight fraction of reinforcement differing in each composites [59]. Stir-casting techniques are also employed for developing hybrid-nanocomposites of aluminium. Suitable reinforcements combined in optimum quantities enhance the mechanical, microstructural, and tribological properties of composites [84]. A summary of the research studies in the stir-casting process of hybrid AA 6061 composites is given in the Table 3.

## 5. AA 6061 Matrix Nanocomposites

Micro-level reinforcements are being commonly used in AMCs. Technological progression in the field of nano sciences makes it possible to employ reinforcements with nano size in MMCs, and the resulting composites are said to be metal matrix nanocomposites (MMNCs). Reinforcement in MMNCs is in the nanometer range (10^−9^ m). The factors such as homogenous distribution, finer size of particles, hardening mechanism, inter-particle spacing, and high temperature thermal stability are the primary reasons for the improvement in the properties of MMNCs [85]. The nano-sized particle-reinforced MMNCs possess superior strength, ductility, and more resistance to wear compared to MMCs reinforced with micro-sized particles. Hence, the former is the better choice of material in terms of material properties, which could have extensive applications in many industrial fields [86]. Researchers have reported that incorporating nano-sized reinforcement particles with aluminium alloy matrix results in superior mechanical, tribological, physical, and interfacial capabilities of the base material.

AA 6061/nano Al_2_O_3_ composites were fabricated by Hamid Reza Ezatpour et al. [71] through the stir-casting method. Al_2_O_3_ particulates were injected into the molten alloy by argon gas along with mechanical stirring. The objective of the study was to examine the effects of addition of nano-sized alumina particles to AA 6061 alloy and the extrusion process on the properties of the composites. Nanocomposites underwent the process of hot extrusion at 550 °C. The weight fractions of nano-Al_2_O_3_ powder injected into the composites were chosen as 0.5, 1 and 1.5 wt %. Yield strength, ultimate strength, and hardness of composites were reported to be higher than that of the alloy matrix in both the as-cast condition and extruded state. It is attributed to refinement in grains, the Hall–Petch mechanism, and particle strengthening effects, which inhibited dislocation motion. For as-cast composites, the yield strength, ultimate strength, and hardness increased with the increase in reinforcement content up to 1 wt %. Beyond this weight fraction, these values showed reduction. The high porosity content and heterogeneous distribution of nanoparticles led to this effect. However, extruded composites showed an increase in yield strength, ultimate strength, and hardness for all samples with an increment in Al_2_O_3_ content. This behavior of extruded samples was explained by the porosity reduction, better homogenous distribution of nanoparticles, enhanced matrix–particle interface bonding, and the improvement of microstructural densification observed after hot extrusion. Therefore, nanocomposites fabricated by stir casting possessed a fine grain microstructure with high porosity. For increasing the reinforcement content, the porosity volume expanded and was decreased with the process of extrusion.

Ultrasonic-assisted stir casting followed by the squeeze-casting (SQC) technique was carried out to fabricate AA 6061/nano Al_2_O_3_ composites [72]. The solidification of the molten material happens in the squeeze-casting process as a consequence of the application of considerable pressure inside a reusable casting die. The Al_2_O_3_ concentration varied from 1 to 3 wt %. A comparison of the mechanical properties of composites fabricated by the squeeze-casting method and ultrasonic stir-casting process were carried out. It was found out that the hardness, tensile, and compressive strength of the SQC composites were higher than that of composites fabricated through the ultrasonic-assisted stir-casting process. Significant grain refinement was also observed in the microstructure of the SQC composites. With an increase in the weight fraction of nano Al_2_O_3_, the material properties of the composites improved. However, the weight fraction greater than 2% is not advised, as the tensile and compressive strength begin to decrease beyond 2 wt %. This impact could have been triggered by the clustering or agglomeration of nanoparticles at higher weight fractions. It was inferred that porosity formation can be greatly reduced by squeeze casting for AA 6061 nanocomposites.

The tribological behavior of AA 6061/nano TiC/graphite hybrid composites made through stir casting was studied by Sozhamannan G. et al. [73]. The matrix contained evenly distributed reinforcement particles of TiC (3 wt %) and graphite (10 wt %). During the wear process, the existence of reinforcements in the matrix limited the plastic deformation, and the rate of wear was observed as 4.7% lower than that of the base alloy. The composite showed a considerable reduction in weight loss during the wear test, which was 23.33% lower than that of the AA 6061 unreinforced matrix.

Stir casting was employed to produce AA 6061 nanocomposites with 1 wt % and 2 wt % of nano-silver (Ag) [74]. It exhibited excellent mechanical properties and wear resistance, compared to conventional aluminium alloy. A fine-grained microstructure, realistic silver nanoparticle distribution, and low porosity were observable in the nanocomposites. It was found that as the mass fraction of nano-Ag particles increased, the properties such as the hardness, density, tensile, and compressive strength of nanocomposite also increased. The hardness value increased from 92 to 98 BHN when reinforcement was increased from 0 to 2 wt %. Tribological properties were improved as the wear resistance and coefficient of friction of the reinforced composites increased with increase in nano-Ag mass fraction. The research showed that the fabricated composite has the potential to replace conventional materials for better properties.

Utkarsh Pandey et al. [46] reported that the uniform distribution of nano-sized particles in the molten material is highly tedious through the stir-casting method, since the reinforcements have a higher surface-to-volume ratio and poor wettability. The ultrasonic vibration is reported to be effective in dispersing nanoparticles in the melt. An ultrasonic vibrating probe is employed in ultrasonic-assisted stir-casting method to disperse the nano-sized particles into the molten pool in a uniform manner. Initially, the probe will be located at three quarters of depth of molten charge and will be triggered to vibrate vigorously. High energy waves generated due to the vibrations could cause the formation of cavitation bubbles in a considerable number. Extreme pressure and temperature will be prevailing inside the bubbles. Then, the spontaneous explosion of bubbles took place because of the difference in pressure. It could break the clusters and agglomerations and uniformly distribute the well mixed nanoparticles within the molten charge. Then, the resulting molten composite will be transferred to a pre-heated mold in order to prevent stress concentration and cooling from taking place at normal atmospheric condition [75]. Ultrasonic-assisted stir casting was employed successfully to fabricate AA 6061-nano Al_2_O_3_ composites with 1.0, 1.5, 2.0, 2.5, and 3.0 weight % of reinforcements. A significant reduction in porosity was observed due to the ultrasonic processing in the stir-casting fabrication route [76].

## 6. Discussion

Various reinforcements are being used with the AA 6061 matrix to form AMCs. SiC, Al_2_O_3_, B_4_C, and TiC particulate reinforcements are very popular reinforcements owing to their excellent ability of composite characteristics improvement. Many other reinforcements such as molybdenum disulfide, glass, iron ore, red-mud, hematite, rutile, steel machining chips, and bamboo charcoal were also used to form AA 6061 AMCs. Stir casting was successful for all the reinforcements discussed in the review, because the uniform distribution of reinforcements in matrix was observed in microstructural evaluations of composites. Excellent bonding exists between matrix and reinforcement in the composites with uniform distribution. The mechanical characterisation of the composites revealed that the tensile strength, compressive strength, and hardness were improved as the weight fraction of reinforcement increased. Wear properties also showed considerable improvement because of the presence of reinforcement particles in composites. Grain size was reduced as a result of reinforcement addition. However, increasing the weight fraction beyond a limit may deteriorate the properties due to increased porosity, agglomeration, and non-homogenous particle distribution at higher reinforcement content.

Two or more reinforcement particles were used in hybrid AA 6061 composites, which resulted in better properties. Reinforcements can be selected based on the specific properties desired. It is evident from the available literature that the incorporation of secondary reinforcements further enhanced the properties of composites. Scientific optimisation can be carried out to find the optimum quantity of each reinforcements and process parameters. Since more than one type of material is reinforced in hybrid composites, each type could contribute particularly to the enhancement of the mechanical properties of the composite. In addition, there are chances that it may reduce another property. This scenario was observed in the AA 6061-alumina-molybdenum disulfide hybrid composite, in which the mechanical properties and wear resistance increased as the alumina content increased. Meanwhile, the tensile strength and hardness of the composite were reduced due to the increment in the weight fraction of MoS_2_ particulates. However, the addition of MoS_2_ improved the wear and friction resistance of the composite. Hence, suitable reinforcements should be combined in optimum quantities to enhance the mechanical, microstructural, and tribological properties of composites. Hybrid AA 6061 composites can be effectively engineered, depending on the applications and required properties. The stir-casting method was found to be well suitable for fabricating the hybrid composite due to porosity-free castings. There has been an increasing trend in the usage of industrial as well as agricultural waste products as dispersed phase. Materials such as fly ash, bamboo charcoal, rice husk, and other industrial as well as agricultural residues are being used. Reinforcement addition should be limited to the optimum weight fraction in order to avoid material defects.

Nanocomposites of AA 6061 produced by stir casting exhibited enhanced mechanical properties. However, limited studies have been found regarding the stir casting of AA 6061 MMNCs. Only 14% of the literature reviewed focussed on nanocomposites of AA 6061. Porosity formation is a major drawback reported in the process. The nano-sized reinforcement particulates have a higher surface-to-volume ratio and poor wettability. Thus, the dispersion of particles in a matrix phase becomes non-uniform. The heterogenous distribution of reinforcement particles in the matrix also affects the properties of composites. When the reinforcement weight fraction is increased beyond a limit, the composite shows a decline in characteristics. Usually, at higher reinforcement content, the clustering and agglomeration of nanoparticles takes place, which affects the properties. The volume of porosity will also increase as the amount of nanoparticulates increases. Hence, the addition of reinforcement should be properly controlled. The available literature suggested that ultrasonic-assisted stir casting followed by squeeze casting can effectively reduce the porosity and increase the homogenous distribution of nanoparticles. Ultrasonic vibration will be very effective in dispersing the nanoparticles uniformly in the matrix. Vigorous vibration generated by the ultrasonic vibrator could be able to inhibit the formation of clusters and agglomeration in composites. High-power ultrasonic vibrations generated by the ultrasonic probe could lead to acoustic streaming and strong cavitation effects. Transient cavitation triggers the breakdown of gas microbubbles near the reinforcement particle clusters to shatter the clustered particles and disperse them uniformly in the molten pool. In addition, acoustic flow, which is the flow of liquid due to the acoustic pressure gradient, causes the stirring to be extremely effective [87]. Squeeze casting of the composite is also greatly recommended for reducing the material defects and improving the mechanical properties. Solidification under pressure would results in fine grained microstructure with homogenous dispersion. Future studies could focus on the fabrication of MMNCs through stir casting combined with squeeze casting, since it proven as an economical as well as effective method of MMNC fabrication.

The weight fraction of reinforcement content has a significant effect on improving the characteristics of the composite material. All the research mentioned in Table 2 substantiates this result. It is evident that the fabricated composites showed better properties than the base material. The properties of AA 6061 alloy were significantly improved by the addition of reinforcements. Generally, as the weight fraction of reinforcement in the composite increases, mechanical properties such as the hardness, tensile strength, and impact strength show improvement. Wear resistance was also improved as the content of reinforcement increases. However, the addition of reinforcement beyond a certain limit may cause a reduction in properties. This was mainly due to the formation of porosity, clusters, and agglomeration at a higher level of reinforcement content. An excellent mixing of matrix and reinforcement phases is necessary to achieve a homogenous distribution of particulates. Ultrasonic-assisted stir casting and squeeze casting can be employed for fabricating composites with better properties and fewer defects.

Microstructural evaluation is also important to determine whether the stir casting of matrix and reinforcements was successful or not. The distribution of reinforcements in the matrix phase needs to be observed through microstructural examination. For the AA 6061 composites discussed in this review, the dispersion of reinforcement is preferred to be uniform. The intra-granular distribution of reinforcements with a clear interface between the matrix phase is needed for the improved properties of AMC. The microstructure is also used to check the presence of undesirable pores, voids, clusters, and agglomerations, which can affect the material properties. It also indicates whether the fabrication through stir casting is effective or not. The reinforcement particles added have a significant role in the solidification of the molten composite and result in grain refinement. As the content of reinforcement particles increased, more nucleation sites were formed, which resulted in finer grains.

**Figure 5:**
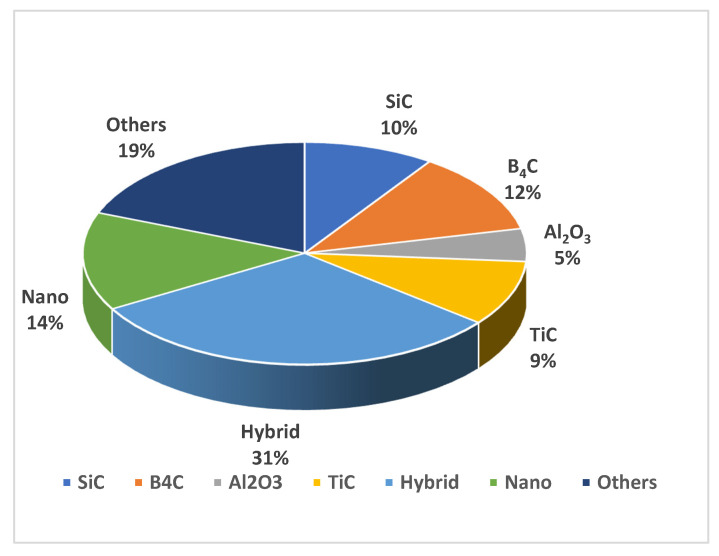
Percentage of research studies related to reinforcement types used in AA 6061 composites.

After reviewing the research works related to AA 6061 metal matrix composites and nanocomposites produced by stir casting, it can be found that the majority of studies investigated the characteristics of composites and the improvement in properties due to reinforcement addition. Researchers paid more attention to the fabrication of hybrid composites through stir casting to obtain enhanced properties, which is attributed to the presence of the secondary dispersed phases. Figure 5 shows the percentages of the research related to reinforcements types used in AA 6061 composites.

About one-third of the research studies were based on hybrid composites, indicating the trend of an increase in the application of hybrid composites. Although SiC, Al_2_O_3_, B_4_C, and TiC had been widely employed as reinforcements in AMCs, now, researchers are more interested in the combination of several reinforcements: 19% of the researches are based on composites with single reinforcement other than SiC, Al_2_O_3_, B_4_C, and TiC. This indicates the emerging trend of using organic as well as inorganic materials as reinforcement in MMCs. It also accounts for the application of industrial and agro residues as reinforcements.

## 7. Conclusion

This review has discussed the production of AA 6061 metal matrix composites by the stir-casting process. Stir casting is identified as the most cost-effective and commercially used fabrication route among various processes. The reviews lead to the following conclusions.

- AA 6061 is one of the most popular aluminium alloys used in various applications. Numerous AA 6061 composites were produced by reinforcing various organic and inorganic materials by the stir-casting method. The composites fabricated showed superior properties to those of the base alloy.
- Stir-casting process parameters such as the speed of the stirrer, stirring time, stirrer blade design, reinforcement size, and melt temperature have a great effect on the AA 6061 composite characteristics. Optimisation can be done to achieve suitable parameters.
- It was reported that reinforcement addition has a significant role in the solidification of the molten composite and results in grain refinement.
- The properties of the AA 6061 composites had strong dependence on the weight fraction of the reinforcement particles. Increasing the weight fraction of reinforcement could improve the mechanical and tribological properties of the composite. However, reinforcement addition beyond a limit would lead to the formation of pores and agglomeration, and it affects the properties.
- Researchers were able to develop economical and potential composite materials by effectively utilising the industrial waste such as fly ash, hence contributing to sustainability.
- Hybrid AA 6061 composites having enhanced properties were developed by stir-casting techniques. The combination of proper reinforcements improved the mechanical, tribological, and corrosion properties of composites.
- Nanocomposites can be also fabricated by stir casting. Limited research studies were done in this area. The high porosity and heterogenous distribution of reinforcement particles were reported in the stir casting of some nanocomposites.
- Future studies could focus on the effective fabrication of MMNCs using stir casting. Ultrasonic-assisted stir casting and squeeze casting were found to be effective in reducing the formation of pores, clusters, and agglomeration. It can lead to the homogenous distribution of reinforcement particulates and improve the mechanical properties.

## Figures and Tables

**Table 1:** Chemical composition of AA 6061 aluminium alloy.

Element	Composition (Mass Percentage)
Al	95.85–98.56
Mg	0.8–1.2
Si	0.4–0.8
Fe	0.0–0.7
Cu	0.15–0.40
Cr	0.04–0.35
Zn	0.0–0.25
Ti	0.0–0.25
Mn	0.0–0.15

**Table 2:** List of studies of AA 6061 composites with different reinforcement types.

No.	Author, Year	Reinforcements Used in AA 6061 Matrix						
		SiC	B_4_C	Al_2_O_3_	TiC	Other	Hybrid	Nano
1.	G. B. Veeresh Kumar et al., 2012 [35]	x						
2.	J. J. Moses et al., 2014 [36]	x						
3.	S. Sivananthan et al., 2020 [37]	x						
4.	N. K. Maurya et al., 2019 [38]	x						
5.	K. Kalaiselvan et al., 2011 [39]		x					
6.	B. Ravi et al., 2015 [40]		x					
7.	D. P. Bhujanga et al., 2018 [41]		x					
8.	B. Manjunatha et al., 2015 [42]		x					
9.	Y. LI et al., 2016 [43]		x					
10.	C. Hima Gireesh et al., 2018 [44]			x				
11.	B. C. Kandpal et al., 2017 [45]			x				
12.	U. Pandey et al., 2017 [46]				x			
13.	S. Gopalakrishnan et al., 2011 [47]				x			
14.	K. Ravi Kumar et al., 2017 [48]				x			
15.	M. S. Raviraj et al., 2014 [49]				x			
16.	M. Marachakkanavar et al., 2017 [50]					x		
17.	M. V. Phanibhushana et al., 2017 [51]					x		
18.	Madhukumar et al., 2018 [52]					x		
19.	K. N. Chethan et al., 2017 [53]					x		
20.	E. Subba Rao et al., 2017 [54]					x		
21.	N. Panwar et al., 2020 [55]					x		
22.	S. R. Prabhu et al., 2019 [56]					x		
23.	M. S. U. Rahman et al., 2018 [57]					x		
24.	K. Baburaja et al., 2016 [58]						x	
25.	S. Ravindran et al., 2019 [59]						x	
26.	V. K. Sharma et al., 2019 [60]						x	
27.	V. K. Sharma et al., 2019 [61]						x	
28.	S. Sarkar et al., 2018 [62]						x	
29.	N. M. Kumar et al., 2018 [63]						x	
30.	G. Pitchayyapillai et al., 2016 [64]						x	
31.	Yashpal et al., 2020 [65]						x	
32.	V. B. Nathan et al., 2020 [66]						x	
33.	C. Elanchezhian et al., 2019 [67]						x	
34.	R. Devanathan et al., 2020 [68]						x	
35.	S. Sachinkumar et al., 2020 [69]						x	
36.	S. J. James et al., 2018 [70]						x	
37.	H. Reza et al., 2014 [71]							x
38.	R. S. Rana et al., 2015 [72]							x
39.	G. G. Sozhamannan et al., 2018 [73]							x
40.	G. Pitchayyapillai et al., 2017 [74]							x
41.	P. Madhukar et al., 2019 [75]							x
42.	K. Sahu et al., 2015 [76]							x

**Table 3:** Summary of research studies in the stir-casting process of hybrid AA 6061 composites.

No.	Authors	Reinforcements and Composition	Remarks
1	Vipin Kumar Sharma et al., 2019 [60]	a. 5 wt % (Al_2_O_3_ + SiC) b. 10 wt % (Al_2_O_3_ + SiC) c. 15 wt % (Al_2_O_3_ + SiC) d. 5 wt % (Al_2_O_3_ + SiC) + 0.5 wt % CeO_2_ e. 10 wt % (Al_2_O_3_ + SiC) + 1.5 wt % CeO_2_ f. 15 wt % (Al_2_O_3_ + SiC) + 2.5 wt % CeO_2_	AA 6061 hybrid AMCs combined with (SiC + Al_2_O_3_) exhibited enhanced mechanical properties with decreased porosity after the incorporation of cerium oxide (rare earth particles). After the successful addition of 2.5 wt % of cerium oxide, the tensile strength increased from 30 to 123 MPa. The micro hardness and Rockwell hardness of the AA 6061 base alloy was improved by 17.02% and 33.80% respectively after the incorporation of 2.5 wt % CeO_2_. Rockwell hardness showed only 16.31% improvement when cerium oxide was not added. The ductility of the hybrid composites was observed to be increased along with the increment in UTS.
2	V.K. Sharma et al., 2019 [61]	a. 5 wt % (Al_2_O_3_ + SiC) b. 10 wt % (Al_2_O_3_ + SiC) c. 15 wt % (Al_2_O_3_ + SiC) d. 5 wt % (Al_2_O_3_ + SiC) + 0.5 wt % CeO_2_ e. 10 wt % (Al_2_O_3_ + SiC) + 1.5 wt % CeO_2_ f. 15 wt %(Al_2_O_3_ + SiC) + 2.5 wt % CeO_2_	The addition of rare earth powder (CeO_2_) oxides to the Al_2_O_3_ and SiC ceramic as an additive is found to be effective in the fabrication of high performing as well as economical AA 6061 hybrid composites. Stir-cast AA 6061 hybrid composites with CeO_2_ demonstrated a decreased level of wear under all test conditions.
3	Siddhartha Sarkar et al., 2018 [62]	Rice husk ash (RHA) + SiC (8 wt % with RHA to SiC ratios 1:4, 2:3 and 0:1)	Microstructure evaluation revealed that a uniform distribution of particulates occurred. The hardness and tensile strength decreased with the increasing wt % of RHA, because of the presence of SiO_2_ having lower hardness and elastic modulus compared to SiC. However, the hardness and tensile strength of the RHA-reinforced products are much higher than that of the non-reinforced AA 6061 alloy, which supports the claim that it can be used as a potential material. AA 6061/RHA/SiC composites showed a greatly reduced porosity level, lower than 2.86% porosity. Hence, these composites can be employed as a lightweight material in engineering applications.
4	N. Mathan Kumar et al., 2018 [63]	Aluminium nitride (AlN) and zirconium boride (ZrB_2_) (0 wt %, 3 wt %, 6 wt %, 9 wt % and 12 wt %)	AA 6061 hybrid composites were effectively fabricated with varied amounts of reinforcements such as 0, 3, 6, 9, and 12 wt %. The particles were well dispersed and were transformed at the time of cooling. The bonding of the matrix and reinforced particles were good; thus, grain refinement was achieved and defects were avoided.
5	G. Pitchayyapillai et al., 2016 [64]	Alumina (4, 8, and 12 wt % of Al_2_O_3_) Molybdenum disulphide (2, 4, and 6 wt % of MoS_2_)	Mechanical properties and wear resistance of hybrid composites increased with an increase in weight fraction of alumina particles. The tensile strength and hardness of the composite were reduced due to the increment in the weight fraction of MoS_2_ reinforcement. However, the addition of MoS_2_ reinforcement in AA 6061/Al_2_O_3_ composites improved the wear and friction resistance of the composite. Metal-to-metal contact was prevented by the stable and MoS_2_ rich mechanically mixed layer, which reduced the wear of the composite.
6	Yashpal et al., 2020 [65]	Alumina (5%) and bagasse ash (8%)	The stir-casting method ensured that the reinforcement distribution was fairly uniform. However, the reinforcement distribution was just up to some degree in the case of large particles. By utilising smaller particle size reinforcements, hybrid composites provided better mechanical properties than the base alloy. However, particle size increment resulted in the reduction of mechanical properties.
7	V. Boobesh Nathan et al., 2020 [66]	Silicon Carbide (2, 4, 6 wt %) Zirconium Dioxide (3 wt %)	Owing to pour-free sound castings, the stir-casting process is found to be well adapted to the hybrid composite fabrication. AA 6061 with 6 wt % SiC and 3 wt % ZrO_2_ hybrid composites showed a 39% increase in hardness, 20.4% improvement in tensile strength, and 24.2% enhancement in compressive strength when compared to virgin material. This had stronger mechanical and metallurgical responses, which recommended this combination for applications in the automobile and aerospace components manufacturing.
8	C. Elanchezhian et al., 2019 [67]	Silicon, zinc, graphite, chromium (0.5, 1, and 1.5 wt % each)	The incorporation of particles such as alumina, silicon, zinc, chromium, etc. in the AA 6061 matrix contributed to the improvement in the hardness, yield strength, and tensile strength, although ductility showed reduction. The presence of graphite in the aluminium matrix improved the tensile strength and elastic modulus, but it reduced the hardness.
9	R. Devanathan et al., 2020 [68]	SiC (10 wt %) Fly ash (10, 12.5, 15, 20 wt %) Coconut shell ash (2.5, 5, 10 wt %)	A rationally uniform distribution of reinforcements was indicated by microstructure and discontinuities were found in the matrix. Composite with 10 wt % SiC, 20 wt % fly ash, and 10 wt % coconut shell ash showed the highest hardness and strength. It is because of the presence and distribution of a higher amount of coconut shell fines within the matrix.
10	Sachinkumar et al., 2020 [69]	SiC (10 wt %) Fly ash (0, 2.5, 5, 7.5 and 10 wt %)	Fly ash and SiC particulates were homogeneously distributed in a matrix of AMCs. There was a clear interface and perfect bonding between the matrix and reinforcements. The inclusion of fly ash up to 7.5 wt % enhanced the UTS and microhardness. When the weight fraction of fly ash was 10%, there was a decrease in the UTS and microhardness, which might be attributed to the improper mixing of reinforcement particles and matrix material at a higher percentage of fly ash.
11	Johny James et al., 2018 [70]	Zirconium dioxide (10%) Al_2_O_3_ (10%)	It was observed to have greater wear resistance and tensile strength than base alloy. Hardness improved by 70% relative to the parent alloy. During the immersion test, the mass loss was found to decrease as the reinforcement content was increased, which was due to the reduction in the exposure of matrix area to the corrosive solution. Additionally, both Al_2_O_3_ and ZrO_2_ demonstrated outstanding resistance to corrosion.

## Data Availability

No new data were created or analyzed in this study. Data sharing is not applicable to this article.
